# Supplementary material for: Time-series transcriptome analysis identified differentially expressed genes in broiler chicken infected with mixed Eimeria species
Source: Front Genet. 2022 Aug 8;13:886781. doi: 10.3389/fgene.2022.886781 (PMC9393255; doi:10.3389/fgene.2022.886781)
Supplement: Supplementary file 2 [file DataSheet1.ZIP › 4dpi_KEGG.Gsea.1625100298584/KEGG_NITROGEN_METABOLISM.html]

Details for gene set KEGG\_NITROGEN\_METABOLISM[GSEA]

|  || Dataset | TMM\_4dpi\_gct\_format\_4dpi\_gct\_format.Class\_4dpi.cls #PC\_versus\_NC.Class\_4dpi.cls #PC\_versus\_NC\_repos |
| Phenotype | Class\_4dpi.cls#PC\_versus\_NC\_repos |
| Upregulated in class | 0 |
| GeneSet | KEGG\_NITROGEN\_METABOLISM |
| Enrichment Score (ES) | -0.63729274 |
| Normalized Enrichment Score (NES) | -1.7884269 |
| Nominal p-value | 0.0026513478 |
| FDR q-value | 0.10718321 |
| FWER p-Value | 0.1988 |
Table: GSEA Results Summary

  

Fig 1: Enrichment plot: KEGG\_NITROGEN\_METABOLISM      
 Profile of the Running ES Score & Positions of GeneSet Members on the Rank Ordered List

  

| SYMBOL | TITLE | RANK IN GENE LIST | RANK METRIC SCORE | RUNNING ES | CORE ENRICHMENT || 1 | CA6 | na | 1214 | 0.599 | -0.0368 | No |
| 2 | GLUL | na | 2018 | 0.420 | -0.0587 | No |
| 3 | CA9 | na | 3421 | 0.230 | -0.1509 | No |
| 4 | ASNS | na | 3568 | 0.211 | -0.1404 | No |
| 5 | AMT | na | 3961 | 0.171 | -0.1547 | No |
| 6 | CA13 | na | 4641 | 0.111 | -0.1994 | No |
| 7 | CA5A | na | 6198 | -0.022 | -0.3267 | No |
| 8 | CPS1 | na | 7092 | -0.097 | -0.3907 | No |
| 9 | GLUD2 | na | 7626 | -0.146 | -0.4194 | No |
| 10 | HAL | na | 8692 | -0.250 | -0.4814 | No |
| 11 | CA4 | na | 10037 | -0.419 | -0.5484 | No |
| 12 | CA2 | na | 10951 | -0.593 | -0.5608 | Yes |
| 13 | GLS2 | na | 11870 | -1.221 | -0.5062 | Yes |
| 14 | CTH | na | 11879 | -1.237 | -0.3739 | Yes |
| 15 | CA7 | na | 11964 | -1.672 | -0.2013 | Yes |
| 16 | CA8 | na | 11981 | -1.909 | 0.0024 | Yes |
Table: GSEA details [plain text format]

  

Fig 2: KEGG\_NITROGEN\_METABOLISM      
 Blue-Pink O' Gram in the Space of the Analyzed GeneSet

  

Fig 3: KEGG\_NITROGEN\_METABOLISM: Random ES distribution      
 Gene set null distribution of ES for **KEGG\_NITROGEN\_METABOLISM**
